# Increased Risk of 90-Day Complications in Patients With Fibromyalgia Undergoing Total Shoulder Arthroplasty

**DOI:** 10.5435/JAAOSGlobal-D-24-00102

**Published:** 2024-05-09

**Authors:** Joshua G. Sanchez, Albert L. Rancu, Fortunay H. Diatta, Anshu Jonnalagadda, Meera M. Dhodapkar, Leonard Knoedler, Martin Kauke-Navarro, Jonathan N. Grauer

**Affiliations:** From the Yale Department of Orthopaedics and Rehabilitation (Mr. Sanchez, Mr. Rancu, Mr. Jonnalagadda, Ms. Dhodapkar, and Dr. Grauer), and the Yale Department of Plastic and Reconstructive Surgery, New Haven, CT (Dr. Diatta, Mr. Knoedler, and Dr. Kauke-Navarro).

## Abstract

**Introduction:**

Anatomic and reverse total shoulder arthroplasties (TSAs) are effective treatment options for end-stage glenohumeral osteoarthritis. Those undergoing TSA may also have fibromyalgia, a musculoskeletal condition. However, the association of fibromyalgia with shorter and longer term outcomes after TSA has not been well characterized.

**Methods:**

Patients undergoing TSA for osteoarthritis indications were identified in the PearlDiver M165 database from January 2016 to October 2022. Exclusion criteria included age younger than 18 years, shoulder infection, neoplasm, or trauma within 90 days before surgery, and inactivity in the database within 90 days of surgery. Patients with fibromyalgia were matched in a 1:4 ratio to patients without based on age, sex, and Elixhauser Comorbidity Index. Ninety-day adverse events were compared using univariable and multivariable analyses. Five-year revision-free survival was compared using the log-rank test.

**Results:**

Of 163,565 TSA patients, fibromyalgia was identified for 9,035 (5.52%). After matching, cohorts of 30,770 non-fibromyalgia patients and 7,738 patients with fibromyalgia were identified. Multivariable analyses demonstrated patients with fibromyalgia were at independently increased odds ratios (ORs) for the following 90-day complications (decreasing OR order): urinary tract infection (OR = 4.49), wound dehiscence (OR = 3.63), pneumonia (OR = 3.46), emergency department visit (OR = 3.45), sepsis (OR = 3.15), surgical site infection (OR = 2.82), cardiac events (OR = 2.72), acute kidney injury (OR = 2.65), deep vein thrombosis (OR = 2.48), hematoma (OR = 2.03), and pulmonary embolism (OR = 2.01) (*P* < 0.05 for each). These individual complications contributed to the increased odds of aggregated minor adverse events (OR = 3.68), all adverse events (OR = 3.48), and severe adverse events (OR = 2.68) (*P* < 0.05 for each). No statistically significant difference was observed in 5-year revision-free survival between groups.

**Discussion:**

This study found TSA patients with fibromyalgia to be at increased risk of adverse events within 90 days of surgery. Proper surgical planning and patient counseling are crucial to this population. Nonetheless, it was reassuring that those with fibromyalgia had similar 5-year revision-free survival compared with those without.

Anatomic and reverse total shoulder arthroplasties (TSAs) have increasingly become common surgical procedures to treat glenohumeral osteoarthritis.^1-3^ However, like other surgical procedures, preexisting patient comorbidities such as fibromyalgia must be considered with surgical decision-making.

Fibromyalgia is a chronic musculoskeletal disorder characterized by generalized pain with reported tenderness, fatigue, sleep disorder, and/or cognitive difficulties.^4,5^ Fibromyalgia presents in middle age and has been reported to affect 2% to 8% of the general population with a female predominance.^4,6,7^ In addition, previous research has shown patients with fibromyalgia experience a substantial health burden due to loss of function, decreased productivity, decreased health-related quality of life, and associated concomitant comorbidities (such as anxiety, depression, and headache).^8,9,10,11,12,13^

With respect to orthopaedic surgery, a previous large database study by Morell et al^14^ showed patients with fibromyalgia who underwent total hip arthroplasty (THA) were at increased risk of longer hospital stays, opioid medication at both 90 days and 1 year postoperatively, and postoperative emergency department (ED) visits compared with non-fibromyalgia patients. Moreover, a prospective observational cohort study of 665 patients demonstrated increased fibromyalgia scores were associated with inferior total knee and hip arthroplasty outcomes even when fibromyalgia scores fell below the threshold for formal diagnosis.^7^ Yet, other studies have found patients with fibromyalgia to be at increased risk of poor postoperative outcomes and increased costs of care after total knee and hip arthroplasty.^8,9,10,11,12^

Despite the correlation of fibromyalgia with outcomes in other orthopaedic procedures, there is a paucity of literature describing the outcomes of patients with fibromyalgia undergoing TSA. A systematic review by D'Onghia et al^15^ demonstrated fibromyalgia to be highly prevalent in upper extremity orthopaedic surgery (ie, 10.1% of shoulder and elbow surgery patients found to have fibromyalgia) and to be associated with increased postoperative opioid consumption. Paralleling this finding, Khazi et al^16^ demonstrated fibromyalgia to be an independent risk factor of opioid use at 1 year after TSA in a large database study of 12,038 TSA patients. Finally, a previous systematic review highlighted the poor quality of available information and noted that while fibromyalgia seemed to negatively affect postoperative surgical outcomes, future studies were warranted to corroborate this link.^17^

Overall, these findings underscore the importance of elucidating the specific postoperative risks associated with patients with fibromyalgia undergoing TSA. As such, this study aimed to evaluate the risks of postoperative adverse outcomes for patients with fibromyalgia relative to matched controls without fibromyalgia who underwent TSA for osteoarthritis indications in a large, national database. We hypothesized that fibromyalgia, as a chronic musculoskeletal disorder, would increase the risk of 90-day complications after TSA.

## Methods

### Study Population

This retrospective matched cohort study used data from the January 2016 to October 2022 M165 PearlDiver Mariner Patient Claims Database (PearlDiver Technologies). The M165 data set is a large, national administrative database that encompasses approximately 165 million patient lives that is commonly used for orthopaedic outcome studies.^18,19,20,21,22,23,24,25,26,27,28,29^ With the deidentified and aggregated nature of the output data, our institutional review board deemed studies using this data set exempt from review.

Patients undergoing TSA (anatomic or reverse) were identified using the Current Procedural Terminology code 23472 and the International Classification of Diseases, 10th revision (ICD-10) procedural codes 0RRJ0JZ, 0RRK0JZ, 0RRJ00Z, 0RRK00Z. Patients undergoing TSA with shoulder osteoarthritis as at least one of the indications on the date of the index procedure were selected with ICD diagnostic coding. Exclusion criteria included age younger than 18 years at the time of TSA, concomitant or up to 90 days before surgery diagnosis of shoulder infection, neoplasm, or fracture, and not being active in the database for at least 90 days postoperatively.

Patients with fibromyalgia were then identified based on ICD-10 code (M79.7) before surgery. Notably, data abstraction began from 2016 based on more specific fibromyalgia coding since the introduction of ICD-10.^30^ Additionally abstracted data included patient age, sex, and Elixhauser Comorbidity Index (ECI—a marker of overall morbidity burden).^31,32^ To account for potential differences in patient inclusion, those with and without fibromyalgia were matched in a 1:4 ratio for the remainder of the analyses.

### Postoperative Adverse Events

Ninety-day incidence of adverse events was identified using methods previously described.^20,22,33,34,35,36,37^ Severe adverse events (SAEs) were defined as at least one occurrence of the following: sepsis, cardiac events, surgical site infection (SSI), deep vein thrombosis (DVT), or pulmonary embolism (PE). Minor adverse events (MAEs) were defined as at least one occurrence of the following: transfusion, acute kidney injury (AKI), wound dehiscence, urinary tract infection (UTI), pneumonia, or hematoma. All adverse events (AAEs) were defined as at least one occurrence of either a severe or minor adverse event.

Ninety-day readmissions were identified based on the “ADMISSIONS” code within the PearlDiver interface using methods previously described.^33,36,38^ Ninety-day postoperative ED visits were identified. However, readmissions and ED visits were not included in the aggregated event groupings. Patients with a coded prescription history of opioids within 90 days before TSA were identified. Hospital length of stay (LOS) was also assessed.

For 5-year revision-free survival analysis, matched patients who were active in the database for at least 5 years after the index procedure were first selected. Then, time from the index TSA to subsequent total shoulder revision surgery was gathered based on Current Procedural Terminology and ICD-10 coding.

### Statistical Analysis

For initial comparison of those without versus with fibromyalgia, continuous variables (age and ECI) were compared using Student *t*-test and sex was compared using Pearson chi-square test. Matching 4:1 was then done based on these variables with the MATCH function directly in the PearlDiver interface. Patient characteristics were then compared as done for the initial comparisons.

For analysis of 90-day complications of the matched cohorts, univariable comparisons were conducted using Pearson chi-square test or Fisher exact test where appropriate. LOS was compared between matched groups using Student *t*-test. Multivariable logistic regression, controlling for patient age, sex, and ECI, was then conducted to determine independent odds ratios (ORs) and 95% confidence intervals (95% CIs) of 90-day adverse events of the fibromyalgia cohort relative to the non-fibromyalgia cohort.

Five-year survival to revision surgery of the matched cohorts was assessed with a Kaplan-Meier survival curve. Those without and with fibromyalgia were compared with a log-rank test, as previously described.^39^

Statistical analysis was conducted using PearlDiver Bellwether software (PearlDiver) and GraphPad Prism 9.4.1 (GraphPad Software). Statistical significance was defined as *P* < 0.05 for all comparisons.

## Results

### Study Population

Overall, 163,565 patients undergoing TSA for osteoarthritis were identified. Of these patients, 9,035 (5.52%) had fibromyalgia. Patients with fibromyalgia tended to be younger (64.9 ± 8.3 versus 69.5 ± 8.4) and more likely to be female (92.3% versus 51.8%) and had more comorbidities (ECI: 8.4 ± 4.0 versus 5.7 ± 3.6) than non-fibromyalgia study subjects (Table 1, *P* < 0.0001 for all).

**Table 1:** Descriptive Characteristics of Adult Patients With and Without Fibromyalgia Who Underwent TSA for Osteoarthritis

Factor	Nonmatched TSA Groups		*P*	Matched TSA Groups (4:1)		*P*
	Nonfibro	Fibro		Nonfibro	Fibro	
Total	154,530	9,035		30,770	7,738	
Age (mean ± SD)	69.5 ± 8.4	64.9 ± 8.3	**<0.0001**	66.2 ± 7.7	66.2 ± 7.7	0.5099
Sex			**<0.0001**			1.0000
Female	80,081 (51.8%)	8,335 (92.3%)		28,040 (91.1%)	7,052 (91.1%)	
Male	74,449 (48.2%)	700 (7.7%)		2,730 (8.9%)	686 (8.9%)	
ECI (mean ± SD)	5.7 ± 3.6	8.4 ± 4.0	**<0.0001**	7.7 ± 3.7	7.7 ± 3.7	0.5038

ECI = Elixhauser Comorbidity Index, Fibro = fibromyalgia, TSA = total shoulder arthroplasty

A 4:1 match controlling for age, sex, and ECI is shown.

Bold represents statistically significant data.

After 4:1 matching based on age, sex, and ECI, there were 30,770 patients without fibromyalgia and 7,738 patients with fibromyalgia (Table 1). Age, sex, and ECI were no longer markedly different between the matched cohorts.

### Postoperative Complications

The incidences of adverse events within 90 days of TSA for those with and without fibromyalgia are shown in Table 2. By univariable analysis, patients with fibromyalgia were more likely to experience AAEs, SAEs, MAEs, sepsis, SSI, cardiac events, DVT, PE, UTI, wound dehiscence, pneumonia, AKI, hematoma, transfusion, and ED visit. Patients with fibromyalgia also demonstrated longer hospital LOS and more preoperative opioid prescriptions within 90 days before surgery. All data are shown in Table 2, with *P* < 0.001 for all.

**Table 2:** Univariable Analyses of 90-Day Complications, Readmissions, Emergency Department Visits, Hospital Length of Stay, and Opioid Prescriptions Within 90 Days Before Surgery

Factor	Nonfibro (n = 30,770)	Fibro (n = 7,738)	*P*
Any adverse events	2,794 (9.1%)	1,891 (24.5%)	**<0.0001**
Severe adverse events	959 (3.1%)	607 (7.8%)	**<0.0001**
Sepsis	268 (0.9%)	207 (2.7%)	**<0.0001**
Surgical site infection	137 (0.4%)	97 (1.3%)	**<0.0001**
Cardiac events	159 (0.5%)	108 (1.4%)	**<0.0001**
Deep vein thrombosis	332 (1.1%)	204 (2.6%)	**<0.0001**
Pulmonary embolism	252 (0.8%)	127 (1.6%)	**<0.0001**
Minor adverse events	2,209 (7.2%)	1,620 (20.9%)	**<0.0001**
Urinary tract infection	1,048 (3.4%)	1,015 (13.1%)	**<0.0001**
Wound dehiscence	98 (0.3%)	90 (1.2%)	**<0.0001**
Pneumonia	531 (1.7%)	437 (5.6%)	**<0.0001**
Acute kidney injury	622 (2.0%)	390 (5.0%)	**<0.0001**
Hematoma	115 (0.4%)	59 (0.8%)	**<0.0001**
Transfusion	152 (0.5%)	65 (0.8%)	**0.0004**
Readmissions	983 (3.2%)	281 (3.6%)	0.0585
ED visit	4,055 (13.2%)	2,563 (33.1%)	**<0.0001**
Hospital length of stay (d)	5.70 ± 5.96	6.63 ± 6.96	**<0.0001**
Preoperative opioids	489 (1.6%)	213 (2.3%)	**<0.0001**

ED = emergency department, Fibro = fibromyalgia

Bold represents statistically significant data.

**Figure 1:**
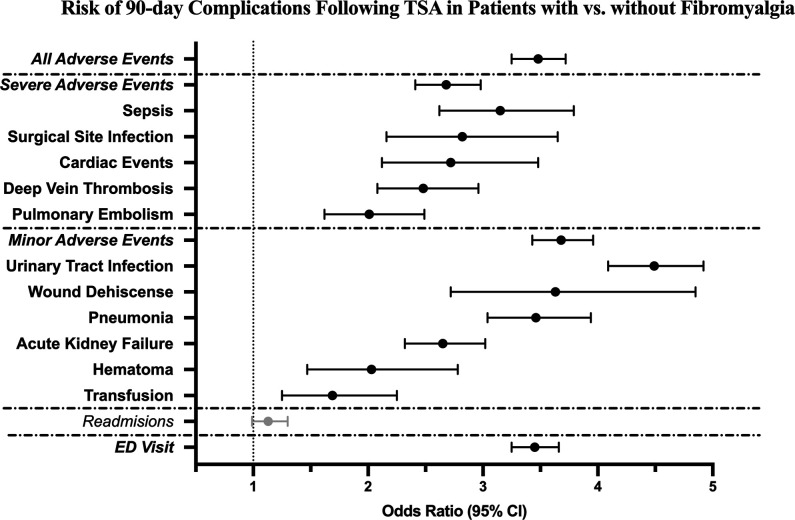
Graph showing forest plot of odds ratios with 95% CIs in the matched fibromyalgia cohort relative to the control cohort. Black bars are statistically significant, whereas gray bars are not. CI = confidence interval, ED = emergency department, TSA = total shoulder arthroplasty

Multivariable analyses demonstrated patients with fibromyalgia were at independently increased ORs for the following individual 90-day adverse outcomes (in decreasing OR order): UTI (OR = 4.49), wound dehiscence (OR = 3.63), pneumonia (OR = 3.46), ED visit (OR = 3.45), sepsis (OR = 3.15), SSI (OR = 2.82), cardiac events (OR = 2.72), AKI (OR = 2.65), DVT (OR = 2.48), hematoma (OR = 2.03), and PE (OR = 2.01) (*P* < 0.05 for each). These individual complications contributed to the increased odds of aggregated MAEs (OR = 3.68), AAEs (OR = 3.48), and SAEs (OR = 2.68) (*P* < 0.05 for each). These results are presented in Table 3 and Figure 1.

**Table 3:** Multivariable Analyses (Controlling for Age, Sex, and Elixhauser Comorbidity Index) of 90-Day Complications and Readmissions of Matched Cohorts

Factor	Fibro OR (95% CI)	*P*
All adverse events	3.48 (3.25-3.72)	**<0.0001**
Severe adverse events	2.68 (2.41-2.98)	**<0.0001**
Sepsis	3.15 (2.62-3.79)	**<0.0001**
Surgical site infection	2.82 (2.16-3.65)	**<0.0001**
Cardiac events	2.72 (2.12-3.48)	**<0.0001**
Deep vein thrombosis	2.48 (2.08-2.96)	**<0.0001**
Pulmonary embolism	2.01 (1.62-2.49)	**<0.0001**
Minor adverse events	3.68 (3.43-3.96)	**<0.0001**
Urinary tract infection	4.49 (4.09-4.92)	**<0.0001**
Wound dehiscence	3.63 (2.72-4.85)	**<0.0001**
Pneumonia	3.46 (3.04-3.94)	**<0.0001**
Acute kidney injury	2.65 (2.32-3.02)	**<0.0001**
Hematoma	2.03 (1.47-2.78)	**<0.0001**
Transfusion	1.69 (1.25-2.25)	**0.0005**
Readmissions	1.13 (0.99-1.30)	0.0679
ED visit	3.45 (3.25-3.66)	**<0.0001**

CI = confidence interval, ED = emergency department, Fibro = fibromyalgia, OR = odds ratio

Bold represents statistically significant data.

**Figure 2:**
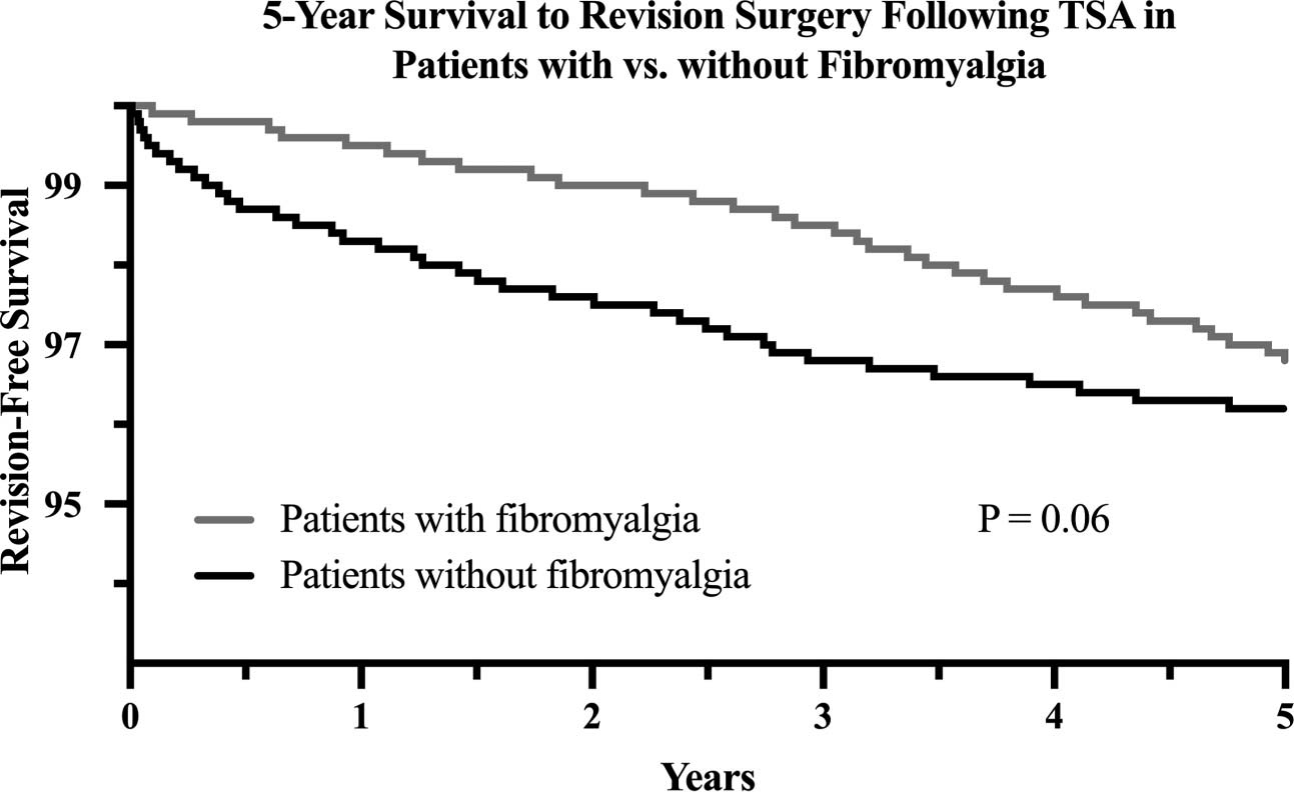
Graph showing Kaplan-Meier curve comparing 5-year revision-free survival in adult patients who underwent total shoulder arthroplasty with fibromyalgia with patients without fibromyalgia. *P* value resulting from a log-rank test is shown.

A 5-year Kaplan-Meier curve displaying the log-rank test results (*P* = 0.06) is shown in Figure 2. No statistical difference was observed in survival to revision surgery between the matched cohorts of patients with versus without fibromyalgia.

## Discussion

To optimize the perioperative care pathway for patients with fibromyalgia considered for TSA, it is crucial to understand associated adverse events. Although previous research has described the risks of patients with fibromyalgia receiving total hip and knee arthroplasty, there had still been a lack of literature explicitly covering the disorder's relationship with TSA.^14,15,17,40-42^ This study characterized the elevated risks of several 90-day complications associated with patients with fibromyalgia undergoing TSA, including infectious, bleeding-related, and venous thromboembolic events, and increased postoperative healthcare utilization.

Fibromyalgia warrants consideration as a potential patient comorbidity as 5.52% of the study population was identified to have the pain disorder. Before matching, those with fibromyalgia were younger, included more women, and had higher comorbidity than those without fibromyalgia undergoing TSA. This aligns with expectations based on previous literature that has characterized the higher prevalence in women, the associated higher comorbidity burden, and younger age at THA for those with fibromyalgia.^4,5,14^ It was based on these differences that matching was done to account for differences in cohort characteristics for this study.

Once matched, multivariate analysis of observed adverse events was conducted to determine outcomes that were at independently greater odds for those with fibromyalgia. Aggregated any, severe, and minor adverse outcomes were of greater odds (OR = 1.77, 1.51, and 2.08, respectively). These increased odds of adverse events after TSA align with studies regarding other orthopaedic procedures.^14,15,42^

In terms of individual adverse events, this study found patients with fibromyalgia to be at greater odds of infectious complications within 90 days of TSA, including sepsis (OR = 2.22), SSI (OR = 2.21), pneumonia (OR = 2.08), UTI (OR = 1.94), and AKI (OR = 1.43). The increased risk of infection is in line with previous studies within large, national databases that described the increased risk of UTI, pneumonia, and acute kidney failure for patients with fibromyalgia undergoing either total hip or knee arthroplasty.^40,41,43^ However, the lack of increased risk of revision surgery for patients with fibromyalgia must be considered because postoperative infection often can be an indication for revision surgery. Overall, these data may help guide perioperative decision-making by increasing awareness and suspicion, especially if symptoms begin to develop, of postoperative infection for this patient population.

Furthermore, patients with fibromyalgia were at increased risk of DVT (OR = 2.48) and PE (OR = 2.01) in this study. Previous research has not previously described an increased risk of thrombotic events associated with fibromyalgia; however, the pathophysiology behind the potential increased risk of thrombotic events may be related to the endothelial dysfunction caused by chronic pain and excessive activation of the sympathetic nervous system in fibromyalgia, as described by Cho et al.^44^ Furthermore, the increased risk of DVT could also be contributed by slower mobilization for this population given chronic pain-related issues, although this may be more relevant to hip or knee arthroplasty surgery. Slower mobilization because of pain-related issues would also track with the previously described association with fibromyalgia and prolonged opioid usage after total hip and knee arthroplasty.^45^

This study also found those with fibromyalgia to be at greater odds of ED visits (OR = 3.45) and longer LOS (6.63 ± 6.96 versus 5.70 ± 5.96 days) than the matched non-fibromyalgia cohort. Critically, preventing ED visits and decreasing LOS are some of the highest effect means for containing costs after joint arthroplasty surgery.^14,46,47^ In line with the results of this study, a previous database study by Morell et al^14^ characterized the association of fibromyalgia with increased LOS and postoperative ED visits after THA. In addition, Nelson et al^42^ found patients with fibromyalgia were at increased risk of 90-day readmission and higher costs after THA in a large database study. These findings show patients with fibromyalgia often require a higher level of healthcare utilization, which should be considered in preoperative planning and patient counseling.

Finally, no difference was found in 5-year survival to revision surgery between the study cohorts. Considering the increased healthcare utilization found within 90 days postoperatively, these data suggest patients with fibromyalgia have a higher risk of healthcare utilization limited to the perioperative period. As such, the diagnosis should not be considered a contraindication to TSA.

There are limitations to consider when interpreting the results of this study. Like other research involving large, administrative databases, this study is limited by the accuracy of the administrative data. Related to TSA, reverse and anatomic TSA were not able to be distinguished. Regarding fibromyalgia, the lack of agreement on fibromyalgia diagnostic criteria combined with the fact that the condition is a diagnosis of exclusion may have affected coding accuracy.^48^ Next, mortality data were not available in the PearlDiver database. Shoulder-specific metrics and patient-reported outcomes were also not able to be assessed. Furthermore, surgeon differences in considering patients with fibromyalgia as surgical candidates were not able to be considered. Finally, radiographic analysis was not conducted as images were not available in the administrative database.

## Conclusion

Overall, this study provides evidence that patients with fibromyalgia undergoing TSA are at increased risk of several adverse events including infectious complications, increased postoperative healthcare utilization, and DVT. However, it is reassuring that 5-year survival to revision surgery was not different between the study groups. Understanding these relative risks associated with patients with fibromyalgia undergoing TSA may help shoulder reconstructive surgeons advance their surgical planning and optimize patient counseling.
